# Survival from brain tumours in England and Wales up to 2001

**DOI:** 10.1038/sj.bjc.6604603

**Published:** 2008-09-23

**Authors:** B Rachet, E Mitry, M J Quinn, N Cooper, M P Coleman

**Affiliations:** 1 Cancer Research UK Cancer Survival Group, Non-Communicable Disease Epidemiology Unit, Department of Epidemiology and Population Health, London School of Hygiene and Tropical Medicine, Keppel Street, London WC1E 7HT, UK; 2 Département d'Hépatogastroentérologie et Oncologie Digestive, Centre Hospitalo-Universitaire Ambroise-Paré, 9 avenue Charles de Gaulle, Boulogne F-92100, France; 3 Social and Health Analysis and Reporting Division, Office for National Statistics (Room FG/114), 1 Myddelton Street, London EC1R 1UW, UK

Brain tumours comprise 2% of all malignant neoplasms in adults in England and Wales, with some 4000 new cases and 3000 deaths each year. Incidence has increased by approximately 25% since 1971 in England and Wales (Quinn *et al*, 2001). Similar increases have been seen in other western countries (Muir *et al*, 1994). Brain tumours are 20–50% more common in men. Incidence was approximately 25% higher in the most affluent fifth of the population in England and Wales than in the most deprived fifth during 1991–1993 (Quinn *et al*, 2001).

The cause of most brain tumours is unknown. Ionising radiation is the only established cause, although some nitrosamines may be a risk factor (Preston-Martin, 1996). Inherited syndromes may account for 5% of cases. Acquired immunosuppression may increase the risk of cerebral lymphoma. Occupations that have been linked to increased risk include the petrochemical and rubber industries, agricultural work, the nuclear industry and work involving exposure to electromagnetic fields (Preston-Martin *et al*, 2006).

Only tumours of the brain explicitly coded as primary, malignant (behaviour code 3) tumours were included here. Patients previously registered with another primary malignancy at any time since 1971 were excluded. Brain tumours coded as benign (behaviour code 0) or of uncertain or unspecified behaviour (behaviour code 1) are often considered together with malignant tumours (Muir *et al*, 1994), and approximately 10 900 such brain tumours were recorded in the National Cancer Registry during the period 1986–1999, approximately 20% of all recorded brain tumours in all regions of England and Wales (data not shown). These tumours were not considered eligible for analysis. It can be difficult to distinguish primary tumours of the brain from metastases of primary tumours in other organs, which are common, but tumours coded as metastatic (behaviour code 6) were excluded. Malignant tumours of the cranial nerves and spinal cord were also excluded.

The survival analyses reported here are based on the data for 37 917 adults (aged 15–99 years) who were registered with a first, primary, malignant tumour of the brain in England and Wales during the period 1986–1999 and followed up to the end of 2001. These patients represented approximately 86% of those eligible for analysis. For 1.9%, the vital status was unknown on 5 November 2002 when the data were extracted for analysis, and they were excluded. Patients whose brain tumour was not their first primary malignancy (1.8%) were also excluded. Most of the other exclusions were for a recorded survival time of zero (date of diagnosis same as date of death; 9.8% of cases). Some of these patients may have died on the day of diagnosis, but many are likely to have been registered from a death certificate only (DCO) (data not shown), and the two groups could not be distinguished in these data. As the date of diagnosis and thus the duration of survival are not available from a death certificate, both categories were excluded. Such patients may well have shorter than average survival (Berrino *et al*, 1995); hence, their exclusion may have caused slight overestimation of overall survival. However, the proportion of DCO cases was similar between deprivation groups and stable over time; hence, their exclusion is unlikely to have caused bias in the estimates of trends in survival, or of socioeconomic gradients.

During the 1990s, some 50% of brain tumours were specified as arising in the frontal, parietal, temporal or occipital lobes, and only 3% arose in the cerebellum or brain stem, but the site was poorly specified or unspecified for 42% of tumours. The proportions are similar to those for the 1980s (Coleman *et al*, 1999).

Gliomas represented 87% of the tumours, mainly malignant glioma (52%) and astrocytoma (30%). Morphology was ill-defined or undefined for approximately 11%.

## Survival trends

**Figure 1:**
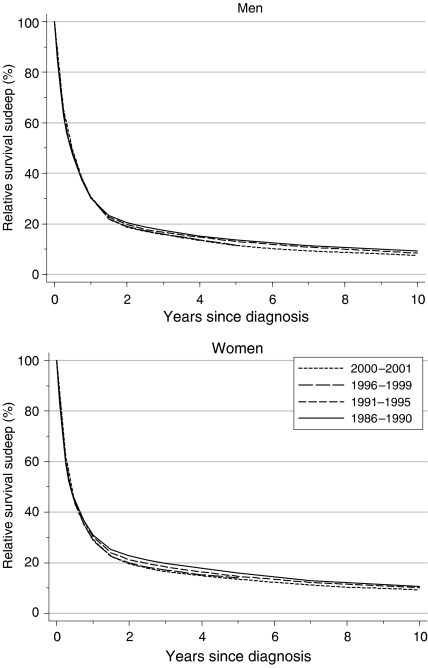
Relative survival (%) up to 10 years after diagnosis by sex and calendar period of diagnosis: England and Wales, adults (15–99 years) diagnosed 1986–1999 and followed up to 2001. Survival estimated with cohort or complete approach (1986–1990, 1991–1995, 1996–1999) or hybrid approach (2000–2001) (see Rachet *et al*, 2008).

Overall survival from tumours of the brain was either stable, or actually fell, in both sexes, between the late 1980s and the late 1990s, reaching 28–30% at 1 year and 12–14% at 5 years (Table 1, Figure 1). The decline in 5-year survival was more marked among men: a statistically significant average change of −3% every 5 years, after adjustment for deprivation. Survival is generally 1–2% higher for women.

Short-term predictions of survival, based on hybrid analysis (Brenner and Rachet, 2004) of the survival probabilities observed during 2000–2001, suggest that this decline may shortly level off, or may even reverse and become positive. Survival would then be expected once again to reach levels similar to those seen for patients diagnosed during 1986–1990, some 10–15 years earlier, at approximately 29–31% at 1 year and 11–14% at 5 years (Table 1).

## Deprivation

For patients diagnosed during 1986–1990, survival up to 10 years after diagnosis was not significantly different among socioeconomic groups, although – as for most other tumours in adults – the general pattern was one of slightly lower survival among patients in the more deprived groups, particularly at 1 year after diagnosis (Table 2).

**Figure 2:**
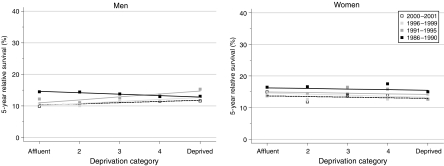
Trends in the deprivation gap in 5-year relative survival (%) by sex and calendar period of diagnosis: England and Wales, adults (15–99 years) diagnosed during 1986–1999 and followed up to 2001.

Among men, the slight but significant decline in 5-year survival (Table 1) was particularly marked among the most affluent groups (Figure 2), leading to an actual reversal of the deprivation gradient during the 1990s, with 5-year survival in the most deprived groups some 3% higher than in the most affluent groups among men diagnosed during 1996–1999 (Table 2). Flattening (or even reversal) of the deprivation gap in survival was observed at all intervals up to 10 years after diagnosis. For 5-year survival, the average change in the deprivation gradient was statistically significant at +2.5% every 5 years in favour of the most deprived groups. No such change occurred among women.

Short-term predictions of survival derived from the data for 2000–2001 suggest that these trends may be levelling off, with little further change in the socioeconomic difference in survival.

## Comment

The absence of any improvement in survival from brain tumours in adults between 1986 and 1999, for either sex, represents a rare exception to the general trend in cancer survival in England and Wales during this period. For brain tumours, the overall trend is in fact towards a decline in survival in both sexes, which is statistically significant for 5-year survival among men (−3% every 5 years). Hybrid analysis suggests that this decline may soon tail off, or even revert to an increase, but the recent decline still stands in sharp contrast to survival trends for brain tumours in both sexes between the early 1970s and the late 1980s; over this 20-year period, there was a steady increase in relative survival of 2–3% every 5 years from approximately 21 to 30% at 1 year and from 9 to 14% at 5 years (Coleman *et al*, 1999).

Among brain tumour patients diagnosed in the 1970s and 1980s in England and Wales, survival at 1 and 5 years was generally lower among more deprived groups, even if the individual differences were small, and not always statistically significant. The flattening of the deprivation gradient in survival for both sexes during the 1990s – and the clear reversal among men – thus provides another striking exception to the general pattern seen for other malignancies.

The trends in survival and in the socioeconomic differences in survival may have a common explanation. The gradual introduction of sensitive new diagnostic techniques, such as computed tomography in the 1970s and magnetic resonance imaging from the 1980s (Husband and Reznek, 2004), as well as stereotactic biopsy, may have led to improved diagnosis, registration and classification of brain tumours, particularly among the elderly (Swerdlow *et al*, 2001). If the general improvement in diagnosis from noninvasive imaging had also been more marked among the higher socioeconomic groups, the downward trend in survival and the reversal of the deprivation gap in survival reported here, could reflect improved diagnosis of lethal tumours through better access to new imaging techniques, especially among more affluent groups (Greenwald *et al*, 1981).

We hypothesised that before widespread access to advanced imaging techniques, some patients presenting as a neurological emergency might have been diagnosed with stroke or cerebral metastases, instead of primary cerebral tumour (MacVicar, 2004). Information on diagnostic investigations for each patient was not available in cancer registry data; however, to explore this possibility, we analysed trends in the incidence of brain tumours by deprivation group and duration of survival. The incidence of brain tumours with survival up to 1 year increased by nearly a third among men in the most affluent groups between 1986 and 1995, but remained stable for men in the most deprived category until 1995. In contrast, trends in the incidence of brain tumours with survival greater than 1 year hardly differed between men in affluent and deprived groups. For women, incidence trends between 1986 and 1999 were very similar in all deprivation groups, and the deprivation gap in survival has not changed significantly over the same time period either. The differential trends in the incidence by deprivation group, lethality and sex seem to be commensurate with the observed trends in survival.

It is thus possible that the apparent decrease in survival from brain tumours in adults of both sexes, and the reversal of the deprivation gap in men, as well as the increase in incidence, especially of patients with poor survival, may be the result of diagnostic exclusion of stroke and cerebral metastases from malignancy in other organs, and improved diagnosis of lethal primary malignancy of the brain. It remains unclear why such a diagnostic shift should affect men more than women.

## Figures and Tables

**Table 1:** Trends in relative survival (%) by sex, time since diagnosis and calendar period of diagnosis: England and Wales, adults (15–99 years) diagnosed during 1986–1999 and followed up to 2001

		**Calendar period of diagnosis^a^**									
		**1986–1990**		**1991–1995**		**1996–1999**		**Average change (%) every 5 years^b^**		**Prediction^c^ for patients diagnosed during 2000–2001**	
**Time since diagnosis**		**Survival (%)**	**95% CI**	**Survival (%)**	**95% CI**	**Survival (%)**	**95% CI**	**Survival (%)**	**95% CI**	**Survival (%)**	**95% CI**
1 year	Men	**30.2**	(29.1, 31.3)	**30.6**	(29.6, 31.6)	**30.4**	(29.3, 31.5)	**−0.7**	(−2.7, 1.4)	**30.7**	(29.1, 32.2)
	Women	**30.9**	(29.6, 32.2)	**30.3**	(29.1, 31.5)	**28.8**	(27.5, 30.1)	**−0.5**	(−2.9, 2.0)	**29.1**	(27.3, 30.9)
5 years	Men	**13.6**	(12.8, 14.5)	**13.0**	(12.3, 13.8)	**11.6**	(10.7, 12.5)	**−3.1** ^**^	(−4.7, −1.4)	**11.4**	(10.4, 12.5)
	Women	**15.9**	(14.9, 17.0)	**14.6**	(13.7, 15.6)	**14.0**	(12.9, 15.1)	**−0.8**	(−2.9, 1.2)	**13.5**	(12.2, 14.9)
10 years	Men	**9.2**	(8.5, 10.0)	**8.4**	(7.8, 9.2)			**−2.0**	(−4.5, 0.4)	**7.5**	(6.6, 8.4)
	Women	**10.7**	(9.8, 11.6)	**10.1**	(9.3, 11.0)			**−0.3**	(−3.4, 2.8)	**9.3**	(8.2, 10.5)

CI=confidence interval.

a Survival estimated with cohort or complete approach (see Rachet *et al*, 2008).

b Mean absolute change (%) in survival every 5 years, adjusted for deprivation (see Rachet *et al*, 2008).

c Survival estimated with hybrid approach (see Rachet *et al*, 2008).

^**^P<0.01.

**Table 2:** Trends in the deprivation gap in relative survival (%) by sex, time since diagnosis and calendar period of diagnosis: England and Wales, adults (15–99 years) diagnosed during 1986–1999 and followed up to 2001

		**Calendar period of diagnosis^a^**									
		**1986–1990**		**1991–1995**		**1996–1999**		**Average change (%) every 5 years^b^**		**Prediction^c^ for patients diagnosed during 2000–2001**	
**Time since diagnosis**		**Deprivation gap (%)**	**95% CI**	**Deprivation gap (%)**	**95% CI**	**Deprivation gap (%)**	**95% CI**	**Deprivation gap (%)**	**95% CI**	**Deprivation gap (%)**	**95% CI**
1 year	Men	**−2.1**	(−5.3, 1.0)	**0.4**	(−2.5, 3.3)	**−0.3**	(−3.5, 2.9)	**1.0**	(−1.4, 3.4)	**−2.4**	(−6.9, 2.1)
	Women	**−2.7**	(−6.4, 1.1)	−3.5*	(−6.8, −0.1)	−4.1*	(−7.8, −0.4)	**−0.8**	(−3.5, 2.0)	**−4.1**	(−9.3, 1.2)
5 years	Men	**−1.8**	(−4.2, 0.6)	3.7**	(1.5, 5.8)	**2.6**	(−0.1, 5.2)	2.5**	(0.6, 4.4)	**1.5**	(−1.5, 4.5)
	Women	**−0.8**	(−3.8, 2.2)	**−0.8**	(−3.3, 1.7)	**−1.4**	(−4.6, 1.9)	**−0.3**	(−2.6, 2.0)	**−0.8**	(−4.7, 3.1)
10 years	Men	**0.6**	(−1.5, 2.7)	2.1*	(0.1, 4.1)			**1.5**	(−1.4, 4.4)	**0.5**	(−2.0, 3.0)
	Women	**0.0**	(−2.6, 2.5)	**−0.4**	(−2.8, 2.0)			**−0.3**	(−3.8, 3.2)	**−0.4**	(−3.7, 3.0)

CI=confidence interval.

a Survival estimated with cohort or complete approach (see Rachet *et al*, 2008).

b Mean absolute change (%) in the deprivation gap in survival every 5 years, adjusted for the underlying trend in survival (see Rachet *et al*, 2008).

c Survival estimated with hybrid approach (see Rachet *et al*, 2008).

^*^P<0.05; ^**^P<0.01.
